# Redefining axillary management by selecting patients for omission of sentinel lymph node surgery in early breast cancer

**DOI:** 10.37349/etat.2026.1002404

**Published:** 2026-10-09

**Authors:** Wiebren Tjalma, Seppe Coenen

**Affiliations:** General Hospital of Aigio, Greece; ^1^Multidisciplinary Breast Clinic, Unit Gynecologic Oncology, Antwerp University Hospital, 2650 Edegem, Belgium; ^2^Department of Obstetrics and Gynecology, Antwerp University Hospital, 2650 Edegem, Belgium; ^3^Faculty of Medicine and Health Sciences, University of Antwerp, 2610 Wilrijk, Belgium

**Keywords:** Breast Neoplasms, sentinel lymph node biopsy, axilla, de-escalation, omission, precision medicine, radiotherapy, treatment outcome

## Abstract

Axillary staging has traditionally been performed routinely in early breast cancer to provide prognostic information and guide adjuvant therapy. In the era of targeted anti-tumor therapy and biology-driven treatment selection, however, the therapeutic relevance of nodal status has diminished in selected patients with clinically node-negative disease. Recent randomized trials, including Sentinel node vs. Observation after axillary UltraSouND (SOUND), INtergroup SEntinel MAmma trial (INSEMA), and Dutch Breast Cancer Research Group (BOrstkanker Onderzoek Groep; BOOG) 2013-08, have demonstrated that omission of sentinel lymph node biopsy (SLNB) in carefully selected patients undergoing breast-conserving surgery with planned whole-breast radiotherapy (WBRT) does not compromise local or distant disease control and rarely alters adjuvant systemic or radiotherapy management. The most robust evidence applies to postmenopausal women aged ≥ 50 years with ductal tumors ≤ 2 cm, grade 1–2, hormone receptor (HR)-positive, human epidermal growth factor receptor 2 (HER2)-negative and clinically node-negative disease. In this biologically favorable population, the therapeutic impact of nodal findings is limited as systemic therapy and radiotherapy decisions are predominantly driven by tumor subtype and genomic risk stratification rather than minimal nodal involvement. Based on evidence from recent randomized trials and current guidelines, this position paper proposes a practical four-step framework for decision-making regarding omission of SLNB. Redefining the axilla in this context represents a precision-oncology approach to surgical de-escalation, aligning local treatment intensity with biological risk while preserving oncologic safety.

## Introduction

For more than 100 years, axillary clearance was the standard in breast cancer care. Only in the last three decades has axillary management evolved from radical surgical clearance to minimally invasive sentinel staging. It is now advancing toward selective omission driven by biological risk stratification and therapeutic relevance. Few areas in surgical oncology illustrate such a sustained recalibration of necessity over such a short time.

The original rationale for axillary dissection was rooted in the Virchow-Halsted concept of centrifugal tumor spread. Breast cancer was viewed as a locoregional disease that disseminated stepwise through lymphatic channels. On this basis, Halsted introduced radical mastectomy with en bloc axillary lymph node removal in 1894, later refining the procedure in the early twentieth century [1, 2]. Axillary lymph node dissection (ALND) was therefore considered essential for cure and remained a cornerstone of treatment for decades.

Understanding of metastasis evolved in parallel with this surgical doctrine. As early as the eleventh century, Avicenna described the invasive and spreading nature of breast cancer [3]. In 1829, Récamier introduced the term “metastasis,” formally recognizing systemic dissemination [4]. Paget’s “seed and soil” hypothesis in 1889 proposed that metastatic spread depends on the interaction between tumor cells and a permissive microenvironment [5, 6]. Halsted interpreted this biology through a linear model of progression, assuming orderly spread from breast to lymph nodes and subsequently to distant organs [1]. However, alternative concepts emerged. Ewing proposed dissemination according to vascular and lymphatic flow patterns [7]. Later, experimental and clinical evidence demonstrated that only a very small fraction of circulating tumor cells formed metastases, a phenomenon termed metastatic inefficiency [5, 8]. Observations of tumor dormancy further challenged the linear progression; delayed metastases without local recurrence were described by Willis and Hadfield, suggesting a temporary mitotic arrest [5, 9]. In the 1960s, Fisher advanced the systemic disease hypothesis, proposing that breast cancer is often disseminated at diagnosis [10]. Hellman later refined this with the spectrum theory, describing metastatic behavior as a biological continuum [11].

Together, these insights progressively weakened the assumption that regional lymph nodes are obligatory gateways to systemic spread. Nodal metastases were increasingly interpreted as markers of tumor biology rather than the source of dissemination. Randomized evidence reinforced this shift. The National Surgical Adjuvant Breast and Bowel Project (NSABP) B-04 trial demonstrated no overall survival advantage for immediate ALND compared with delayed intervention [12, 13]. Some node-negative patients developed distant metastases, while some node-positive patients did not. These findings altered the perceived role of the axilla: its prognostic value remained, but its therapeutic indispensability was questioned.

The introduction of sentinel lymph node biopsy (SLNB) marked the next decisive transformation. Giuliano and colleagues demonstrated that targeted removal of the first draining lymph node could accurately stage the axilla [14]. Randomized trials, including NSABP B-32 and Axillary Lymphatic Mapping Against Nodal Axillary Clearance (ALMANAC), confirmed equivalent survival and regional control compared with ALND, with substantially lower morbidity [15–17]. SLNB replaced routine dissection in clinically node-negative disease.

The axilla thus transitioned from a therapeutic battlefield to a staging field. Further de-escalation followed in patients with limited nodal involvement. The International Breast Cancer Study Group 23-01 (IBCSG 23-01) trial demonstrated that completion of ALND could be safely omitted in patients with sentinel node micro-metastases [18, 19]. ACOSOG Z0011 extended this principle to selected patients with limited macro-metastatic disease undergoing breast-conserving surgery with whole-breast radiotherapy (WBRT) [20]. In parallel, the AMAROS trial showed that axillary radiotherapy provided regional control equivalent to ALND with lower morbidity [21].

These findings were reinforced by additional studies. The OTOASOR trial confirmed that axillary radiotherapy yields comparable long-term regional control to ALND in patients with limited sentinel node involvement [22]. The SENOMAC trial further broadened the evidence base, including patients with T1–T3 tumors and both breast-conserving surgery and mastectomy. Omission of completion ALND in patients with one or two positive sentinel nodes did not compromise recurrence-free survival [23].

Together, these studies showed that low-volume nodal disease can be safely managed without extensive axillary surgery when effective systemic therapy and radiotherapy are applied. As nodal involvement became increasingly a marker of tumor biology rather than a therapeutic target, the role of routine surgical staging itself came into question.

Initial support for omission of axillary surgery came from observational studies and the Society of Surgical Oncology Choosing Wisely recommendations in women over 70 years with low-risk disease [24–26]. This naturally led to the question of whether SLNB could also be omitted in other clinically node-negative patients with favorable tumor biology.

This question has now been addressed in three contemporary randomized trials: SOUND, INSEMA, and Dutch Breast Cancer Research Group (BOrstkanker Onderzoek Groep; BOOG) 2013-08 [27–30]. The SOUND trial demonstrated that omission of SLNB in patients with tumors ≤ 2 cm and a negative axillary ultrasound did not compromise distant disease-free survival (DFS) [27]. INSEMA expanded eligibility to tumors up to 5 cm and confirmed non-inferior invasive DFS, with only a small absolute increase in axillary recurrence [28]. The BOOG 2013-08 study provided additional supportive evidence in a representative breast-conserving population. Results have been presented at the San Antonio Breast Cancer Symposium 2026; however, full peer-reviewed publication is not yet available [29, 30]. These findings must be interpreted within the context of modern systemic therapy. In hormone receptor (HR)-positive, human epidermal growth factor receptor 2 (HER2)-negative disease, genomic assays frequently guide chemotherapy decisions independently of limited nodal involvement [31, 32]. Similarly, in HER2-positive and triple-negative disease, systemic therapy is primarily determined by tumor biology. Consequently, nodal status no longer changes adjuvant treatment in a substantial proportion of patients with early breast cancer. The available evidence provides the basis for omission of SLNB, but careful patient selection according to current guidelines remains essential for its safe implementation [33].

Axillary surgery has therefore evolved through distinct phases: radical clearance, sentinel staging, selective omission of completion dissection, and now consideration of complete omission in carefully defined patients. The SLNB procedure itself has also evolved. Indo-cyanine green (ICG) fluorescence provides high sentinel lymph node detection rates and represents an effective alternative to conventional mapping techniques. However, improvements in tracer technology do not change the principles of patient selection for omission of SLNB [34]. This progression reflects a broader oncologic principle: treatment intensity should correspond to therapeutic consequence.

With advances in precision oncology, the axilla is no longer an obligatory staging structure for every patient but a biological checkpoint whose assessment should be guided by its impact on treatment decisions. The challenge is to identify the patients in whom SLNB can be safely omitted. This position paper proposes a practical four-step framework to support that decision in daily clinical practice.

## Scope and definitions

The historical evolution of axillary management leads to a straightforward clinical question: “In which patients can SLNB be safely omitted?” This position paper focuses on the patient population in whom omission is currently supported by randomized evidence. The proposed framework applies to patients with invasive breast cancer undergoing upfront breast-conserving surgery followed by planned WBRT. This treatment context is essential, as incidental irradiation of the lower axilla likely contributes to the low regional recurrence rates observed in the omission trials [20]. Patients should have a clinically node-negative axilla confirmed by negative preoperative axillary ultrasound; suspicious lymph nodes require image-guided biopsy before omission of SLNB is considered. The guiding principle is simple: SLNB should be omitted only when nodal information is unlikely to change systemic therapy or radiotherapy planning.

## Methods

A literature search was performed using PubMed/MEDLINE to identify relevant publications up to May 2026. Current international guidelines, including those from ESMO, NCCN, ASCO, and EUSOMA, were also reviewed where relevant. Additional references were identified from the reference lists of relevant publications. Randomized controlled trials, prospective studies, meta-analyses, and international guidelines on axillary staging and omission of SLNB formed the basis of this narrative review. Historical landmark studies were included to provide the necessary background. Conference abstracts were generally excluded, with the exception of the BOOG 2013-08 trial, because no full peer-reviewed publication is currently available.

## Clinical settings where omission of axillary surgery is evidence-based

### Comparative framework and external validity

Within this framework, the oncologic safety of SLNB omission has been evaluated in three contemporary randomized non-inferiority trials: SOUND, INSEMA, and BOOG 2013-08 [27–30]. Key trial characteristics and outcomes are summarized in Tables 1 and 2.

**Table 1:** The main differences between the trials relate to selection criteria.

**Parameter**	**SOUND**	**INSEMA**	**BOOG 2013-08**
Study design	Randomized Non-inferiority	Randomized Non-inferiority	Randomized Non-inferiority
Patients analyzed	1,405 (ITT)	4,858 (PP)	1,572 (PP)
Tumor size	≤ 2 cm	≤ 5 cm	≤ 5 cm
cT1 (≤ 2 cm)	100%	80%	83%
cT2 (2–5 cm)	0%	20%	17%
Clinical nodal requirement	cN0	cN0	cN0
Axillary imaging requirement	Mandatory negative ultrasound; biopsy if suspicious	Per institutional standard	Per national standard
Median age	60 years	62 years	61.5 years
HR+/HER2−	87.8%	95.2%	86.8%
HER2-positive	6.8%	3.6%	7.4%
Triple-negative	5.4%	1.2%	5.8%
Grade 1–2	80%	85–90%	82%
Surgical backbone	BCS + WBRT	BCS + WBRT	BCS + WBRT

SOUND: Sentinel node vs. Observation after axillary UltraSouND; INSEMA: INtergroup SEntinel MAmma trial; BOOG: Dutch Breast Cancer Research Group (BOrstkanker Onderzoek Groep); ITT: intention-to-treat protocol; PP: per-protocol; HER2: human epidermal growth factor receptor 2; HR: hormone receptor; BCS: breast conservative surgery; WBRT: whole-breast radiotherapy.

**Table 2:** Absolute oncologic safety and therapeutic yield of SLNB, oncologic outcomes (consistent across trials).

**Outcome**	**Result**	
DFS (omission vs. SLNB)	Non-inferior	
Absolute DFS difference	< 1%	
Axillary recurrence (omission)	< 2%*	
Absolute increase vs. SLNB	< 1%	
Distant recurrence	No difference	
Overall survival	No difference	
**Therapeutic yield**		
Study	SOUND	INSEMA/BOOG
Estimated % patients with ≥ 1 adjuvant treatment change**	2–6%	3–9%
Dominant driver	CDK4/6 eligibility	CDK4/6 eligibility

SLNB: sentinel lymph node biopsy; DFS: disease-free survival; SOUND: Sentinel node vs. Observation after axillary UltraSouND; INSEMA: INtergroup SEntinel MAmma trial; BOOG: Dutch Breast Cancer Research Group (BOrstkanker Onderzoek Groep); CDK4/6: cyclin-dependent kinase 4 and 6. *: Axillary recurrence rates varied across trials: approximately 0.4–0.6% in SOUND and 1–1.5% in INSEMA; full peer-reviewed data for BOOG 2013-08 are not yet available; **: based on Wanis et al. [35]; dependent on CDK4/6 uptake and radiotherapy practice.

These trials are largely comparable in design and patient population. All included patients with early-stage, clinically node-negative breast cancer undergoing breast-conserving surgery with planned WBRT. All were conducted in an era in which systemic therapy is increasingly guided by tumor biology rather than anatomical nodal staging [31, 32].

The enrolled populations were predominantly postmenopausal and were enriched for estrogen receptor-positive and HER2-negative tumors, mainly luminal A and B subtypes. HER2-positive and triple-negative disease were minimally represented, indicating that the evidence base for omission primarily applies to biologically favorable tumors [27, 28]. Accordingly, extrapolation to HER2-positive or triple-negative disease should be made with caution.

The main differences between the trials relate to selection criteria (Table 1). SOUND represents the most restrictive cohort, limited to tumors ≤ 2 cm with mandatory negative axillary ultrasound and biopsy of suspicious nodes [27]. This closely reflects a screening-detected population, in which approximately 75% of tumors are ≤ 20 mm and around 75% of patients are node-negative at final pathology [36].

In contrast, INSEMA and BOOG 2013-08 included tumors up to 5 cm and applied less stringent imaging requirements, thereby encompassing a broader clinical spectrum [28–30]. The principal structural distinction between SOUND and the other trials lies in tumor size restriction (T1-only vs. T1 & T2), which directly influences expected nodal burden and therapeutic reclassification yield.

### Limitations of the randomized evidence

The available randomized trials provide consistent results, but several limitations should be considered. The eligibility criteria, use of preoperative axillary ultrasound, and tumor size differed between SOUND, INSEMA, and BOOG 2013-08, limiting direct comparison between the studies. Follow-up is still relatively short, particularly for HR-positive breast cancer, where late recurrences remain relevant. In addition, HER2-positive and triple-negative breast cancers were underrepresented. Finally, the BOOG 2013-08 trial has so far only been presented at a scientific meeting and has not yet been fully published in a peer-reviewed journal. These limitations should be considered when applying the results in clinical practice.

### Absolute risk asymmetry and therapeutic yield

Table 2 integrates absolute oncologic outcomes with modeled therapeutic yield, illustrating a consistent dissociation between oncologic safety and clinical utility of SLNB.

Across all trials, omission of SLNB is associated with a very small absolute increase in axillary recurrence, generally below 1%, without any detectable impact on distant recurrence or overall survival [27–30]. The prevented event, an isolated axillary recurrence, is therefore uncommon (generally < 2%) and, in most cases, salvageable. Assuming an excess axillary recurrence risk of approximately 0.5–1.0%, the number needed to treat (NNT) with SLNB to prevent one such event is in the range of 100–200 patients.

In parallel, the therapeutic yield of SLNB remains limited. The clinically relevant question is not nodal positivity per se, but whether nodal information alters adjuvant management. Modeled decision-consequence analyses indicate that SLNB changes at least one component of adjuvant therapy in approximately 2–9% of patients, largely driven by eligibility for cyclin-dependent kinase 4 and 6 (CDK4/6) inhibitors and, to a lesser extent, regional radiotherapy adaptation (Table 2) [35]. The harm profile follows a different scale. Although less morbid than ALND, SLNB is associated with clinically relevant arm morbidity, including lymphedema, sensory disturbance, and functional impairment, in approximately 5% of patients [16, 17], corresponding to a number needed to harm (NNH) of 20.

Taken together, this results in a marked absolute risk asymmetry. More than 100 patients need to undergo SLNB to prevent a single, typically salvageable regional recurrence, while the number needed to harm is 20. At the same time, fewer than 1 in 10 patients will have any meaningful change in adjuvant treatment as a result of nodal staging.

### Number Needed to Stage (NNS): quantifying procedural yield

To quantify the clinical value of nodal staging, we use the NNS, defined as the number of SLNB procedures required to identify one additional patient whose adjuvant treatment would change based on nodal information. The NNS is not intended as a novel mathematical concept, but as a simple clinical metric to quantify the procedural yield of SLNB in different biological subgroups.

Because therapeutic reclassification is primarily driven by eligibility for CDK4/6 inhibitors, the NNS is determined by the proportion of patients in whom nodal status changes treatment. Across contemporary omission-eligible populations, this proportion remains limited [35, 37]. In cT1 patients, nodal staging identifies additional patients eligible for CDK4/6 inhibitors (Table 3). For ribociclib, this is approximately 8% of patients, corresponding to an NNS of 13. For abemaciclib, this decreases to approximately 2%, corresponding to an NNS of 45–48. These differences show that the procedural yield of SLNB depends on the systemic treatment criteria being applied. The NNS should be regarded as a pragmatic clinical metric rather than a fixed value. Because reimbursement policies, indications for systemic therapies, and treatment guidelines vary between healthcare systems, the NNS may differ across countries and over time. Even under maximal CDK4/6 uptake, a substantial number of SLNB procedures are required to identify one additional patient eligible for CDK4/6 inhibitor treatment.

**Table 3:** NNS to identify one additional patient eligible for CDK4/6 inhibitor.

**Clinical scenario**	**CDK4/6 strategy**	**Eligibility dependent on LN status**	**NNS**
T1	Ribociclib	7.5–8.1%	13
	Abemaciclib	2.1–2.2%	45–48

The estimates presented are derived from published data [35, 37]. For abemaciclib, the estimates are based on FDA-approved eligibility criteria. They are intended to illustrate the potential clinical implications of nodal staging. They have not been prospectively validated and should not be interpreted as predictive estimates. NNS: number needed to stage; CDK4/6: cyclin-dependent kinase 4 and 6; LN: lymph node.

### Integrated interpretation

This imbalance fundamentally reframes the role of SLNB in omission-eligible populations. The procedure no longer functions primarily as a decision-defining intervention, but as a low-yield staging test with limited therapeutic consequence.

In biologically favorable, clinically and radiologically node-negative patients undergoing breast-conserving surgery with planned whole breast radiotherapy, the balance between benefit and harm increasingly supports selective omission rather than routine staging.

### Boundaries of current evidence

Despite robust randomized data, omission of SLNB must be applied within clearly defined clinical boundaries. De-escalation is evidence-based only when the treated population reflects the phenotype of the randomized cohorts. Extrapolation beyond this envelope requires caution.

Age represents an important contextual factor. Across SOUND, INSEMA, and BOOG, the median age approximated the early sixties, and the majority of patients were postmenopausal [27–30]. Younger patients were underrepresented. Median age, however, should not be interpreted as a biological threshold. Younger patients have a higher prevalence of aggressive tumor biology, longer life expectancy, and greater cumulative exposure to recurrence risk. Consequently, even small absolute differences in regional control may carry greater long-term relevance. Omission is therefore most robustly supported in postmenopausal patients, while application in younger individuals requires individualized consideration.

Tumor grade constitutes a second boundary. Although grade 3 tumors were not excluded, they represented a minority in all trials [27–30]. The dominant phenotype was grade 1–2 luminal disease. Higher-grade tumors are associated with increased proliferative activity and systemic relapse risk. In this context, proliferative markers such as Ki-67 proliferation index, although not systematically reported in the omission trials, may provide additional biological granularity [38]. A high Ki-67 proliferation index may identify tumors with more aggressive behavior within otherwise luminal subtypes. While this does not automatically mandate nodal staging, it may reduce confidence in omission, particularly when combined with other high-risk features. Accordingly, omission in grade 3 or highly proliferative tumors (Ki-67 ≥ 20 %) should be individualized rather than routine.

Tumor biology further defines the safe envelope. The randomized evidence is strongest in HR-positive, HER2-negative disease. HR positivity was primarily defined by estrogen receptor expression. Progesterone receptor status was not a selection criterion and was not specifically evaluated as a marker of more aggressive luminal biology. HER2-positive and triple-negative subtypes were included but constituted only a small proportion of trial populations [27–30]. Although systemic therapy in these subtypes is primarily biology-driven, their patterns of relapse differ from luminal disease. HER2 overexpression is observed in approximately 30–35% of cases of ductal carcinoma in situ (DCIS), substantially more frequently than in invasive breast cancer, where HER2 positivity is found in approximately 13–15% of cases [39, 40]. This nearly twofold higher prevalence suggests that HER2 amplification is an early event in breast carcinogenesis. HER2-positive DCIS has been associated with an increased risk of ipsilateral local recurrence, particularly following breast-conserving surgery [40]. Nevertheless, routine assessment of HER2 status in DCIS is not currently recommended for treatment decision-making, because anti-HER2 therapy has not demonstrated sufficient clinical benefit in this setting [41]. Consequently, HER2 expression in DCIS is currently regarded as a biological and prognostic marker rather than a therapeutic biomarker. The omission paradigm is therefore most securely anchored in HR+/HER2− tumors and becomes progressively less certain in biologically aggressive subtypes. The current evidence is limited to the patient populations included in the randomized trials and should not be extended to other groups without caution. Evidence remains limited for younger patients and for biologically aggressive subtypes, including HER2-positive and triple-negative breast cancer, which were underrepresented in the available trials. In addition, omission of SLNB should only be considered after standardized preoperative assessment of the axilla, preferably including axillary ultrasound. This further supports the need for standardized reporting of imaging-confirmed nodal status when selecting patients for omission of SLNB [42].

Tumor size also influences external validity. SOUND restricted inclusion to tumors ≤ 2 cm (T1), whereas INSEMA and BOOG extended eligibility to T2 disease [27–30]. Although non-inferiority was maintained, the density of evidence decreases with increasing tumor burden. Larger tumors are associated with higher rates of occult nodal involvement and may more frequently influence radiotherapy planning. Omission in T2 disease is therefore acceptable in the presence of otherwise favorable biology but requires more nuanced multidisciplinary judgment.

The surgical and radiotherapeutic context is critical. All omission trials were conducted in patients with NST (No Special Type) breast cancer undergoing breast-conserving surgery followed by WBRT. Incidental irradiation of the lower axilla likely contributes to the low regional recurrence rates observed [21, 43]. Patients undergoing mastectomy without planned radiotherapy were not adequately represented. Omission should therefore not be generalized to surgical settings lacking axillary radiation exposure.

Finally, omission is inappropriate when nodal status has direct therapeutic consequences. If lymph node involvement would alter eligibility for systemic escalation, such as CDK4/6 inhibition, chemotherapy, or influence regional nodal irradiation, SLNB retains clinical value. The omission strategy is predicated on therapeutic independence from nodal information; when this condition is not met, staging remains justified.

Taken together, the strongest evidence supports omission in a clearly defined phenotype: postmenopausal patients; HR+/HER2− tumors; predominantly grade 1–2 and low proliferative biology; T1 and carefully selected T2 lesions; clinically and sonographically node-negative axilla; breast-conserving surgery with planned WBRT; and systemic therapy decisions driven primarily by tumor biology rather than nodal burden.

Outside this phenotype, omission should not be routine. It should be the result of individualized multidisciplinary evaluation in which biological risk, life expectancy, radiotherapy strategy, and patient preference are carefully weighed. These boundaries are not restrictive, but operational: they define when omission is proportionate. The next step is to translate this evidence-based framework into daily clinical decision-making.

## Clinical implementation for tomorrow: therapeutic proportionality in axillary decision-making

The randomized evidence is mature. The guidelines are converging. The remaining question is no longer whether omission of SLNB is oncologically safe in selected patients, but how to implement this safely and consistently in clinical practice [27–30].

Clinical implementation must be grounded in one guiding principle: therapeutic proportionality. Axillary surgery should be performed only when the information obtained is expected to alter management in a way that improves outcome. When nodal information does not change systemic therapy, does not meaningfully alter radiotherapy planning, and does not improve survival, surgical staging becomes difficult to justify as routine practice.

From tomorrow onward, axillary decision-making can follow a structured sequence (Figure 1).

**Figure 1:**
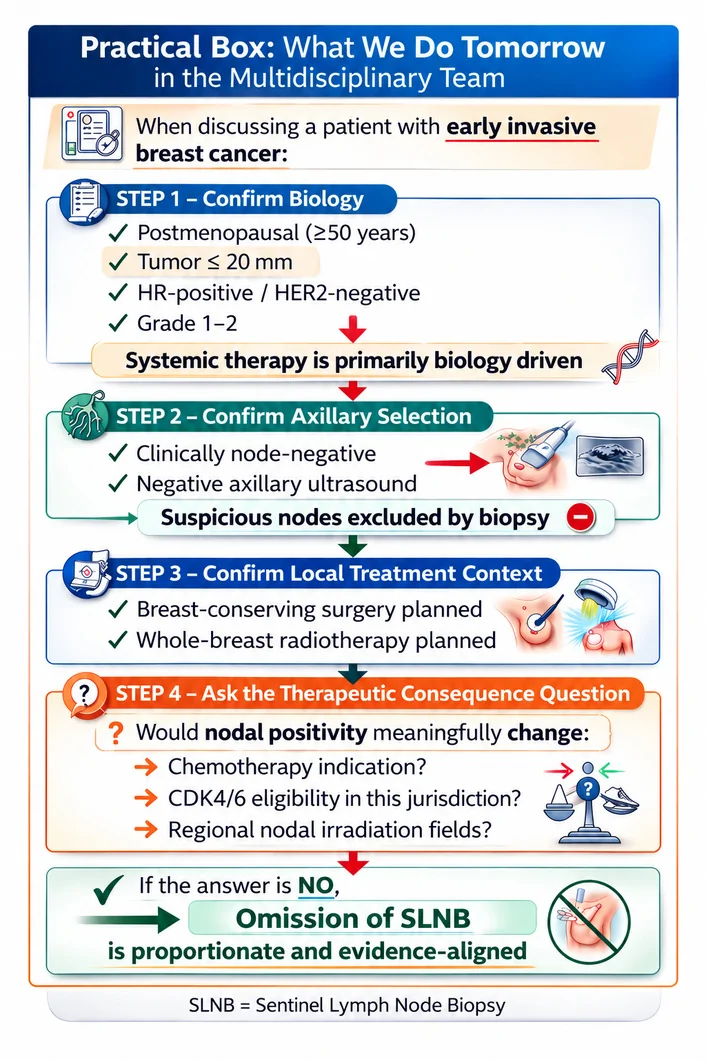
**A practical four-step framework for decision-making regarding omission of SLNB.** HR: hormone receptor; HER2: human epidermal growth factor receptor 2; SLNB: sentinel lymph node biopsy.

First, confirm the biological phenotype. Is the patient postmenopausal? Is the tumor HR-positive/HER2-negative and of low to intermediate grade? Are clinicopathologic or genomic features such that systemic therapy is primarily biology-driven rather than node-driven? If the systemic treatment plan would not change with limited nodal involvement, the therapeutic dependence on SLNB is already weakened.

Second, confirm axillary selection. Is the axilla clinically node-negative and sonographically negative? Suspicious findings should be excluded by biopsy when appropriate. In an imaging-defined node-negative setting, the residual probability of clinically meaningful nodal burden is low, narrowing the incremental staging yield.

Third, define radiotherapy intent. Is WBRT planned? If so, incidental low-axillary coverage contributes to regional control. In contrast, omission cannot be generalized to mastectomy without radiotherapy.

Fourth, assess therapeutic consequence explicitly. Would nodal positivity trigger chemotherapy escalation? Alter regional nodal irradiation? Unlock CDK4/6 inhibitor eligibility? If the answer is “unlikely,” omission is proportionate.

This approach transforms SLNB from a reflex into a decision. It shifts the axilla from an obligatory staging field to a conditional decision node. Implementation does not require structural change, but explicit documentation. A concise multidisciplinary statement documenting clinical node-negative status, negative imaging, biology-driven systemic planning, radiotherapy intent, and informed patient consent is sufficient to operationalize omission safely. Patients should be engaged transparently. The discussion is not about reducing care, but about avoiding low-yield intervention. The trade-off should be communicated in absolute terms: a very small increase in isolated regional recurrence risk, no demonstrated survival disadvantage, and a measurable reduction in arm morbidity.

Clinical implementation is therefore neither abandonment nor enthusiasm-driven de-escalation. It is calibrated restraint. In biologically favorable, imaging-negative, postmenopausal patients undergoing breast-conserving surgery with planned WBRT, omission of SLNB is not experimental. It is evidence-aligned. Outside this phenotype, staging remains appropriate. The paradigm shift is subtle but profound: the axilla is no longer interrogated because it can be staged, but only when staging is expected to change management. That is therapeutic proportionality.

## Conclusion

The routine use of SLNB in all clinically node-negative invasive breast cancers is no longer proportionate to its clinical yield.

Randomized evidence from SOUND, INSEMA, and BOOG 2013-08 demonstrates that omission of SLNB in carefully selected patients undergoing breast-conserving surgery with planned WBRT does not compromise distant disease control and results in only a minimal absolute increase in axillary recurrence, without survival disadvantage. When interpreted in absolute terms, the clinical benefit of SLNB is limited: many patients must undergo staging to prevent a single, typically salvageable regional event, while a smaller number experience avoidable morbidity. At the same time, nodal information alters adjuvant treatment in only a minority, largely depending on systemic therapy frameworks such as CDK4/6 inhibitor use. De-escalation therefore represents not omission of care, but refinement of intent. The axilla should no longer be staged by default, but only when nodal information is expected to change management.

## Abbreviations

ALMANAC: Axillary Lymphatic Mapping Against Nodal Axillary Clearance
ALND: axillary lymph node dissection
BOOG: Dutch Breast Cancer Research Group (BOrstkanker Onderzoek Groep)
CDK4/6: cyclin-dependent kinase 4 and 6
DCIS: ductal carcinoma in situ
DFS: disease-free survival
HER2: human epidermal growth factor receptor 2
HR: hormone receptor
IBCSG 23-01: International Breast Cancer Study Group 23-01
ICG: indo-cyanine green
INSEMA: INtergroup SEntinel MAmma trial
NNH: number needed to harm
NNS: number needed to stage
NNT: number needed to treat
NSABP: National Surgical Adjuvant Breast and Bowel Project
SLNB: sentinel lymph node biopsy
SOUND: Sentinel node vs. Observation after axillary UltraSouND
WBRT: whole-breast radiotherapy

## Declarations

### Copyright

© The Author(s) 2026.
